# Prophylactic and therapeutic CSC-based vaccination reduced tumor growth, metastasis and enhanced survival in mouse model of breast cancer

**DOI:** 10.1186/s13058-026-02242-7

**Published:** 2026-02-19

**Authors:** Masoumeh Dehghan Manshadi, Farideh Hashemi, Sadegh Safaei, Hannaneh Golshahi, Mahmood Bozorgmehr, Leila Eini, Mohammad Reza Bolouri, Zahra Madjd, Roya Ghods

**Affiliations:** 1 Oncopathology Research Center, Iran University of Medical Sciences, Tehran, Iran; 2 Department of Molecular Medicine, Faculty of Advanced Technologies in Medicine, Iran University of Medical Sciences, Tehran, Iran; 3 Nanobiotechnology Research Center, Avicenna Research Institute, ACECR, Tehran, Iran; 4 Department of Veterinary Basic Sciences, SR.C., Islamic Azad University, Tehran, Iran; 5 Department of Immunology, Iran University of Medical Sciences, Tehran, Iran

**Keywords:** Cancer stem cells (CSCs), Spheroid formation, Vaccine, Metastasis, Prophylactic vaccination, Therapeutic vaccination, Breast cancer

## Abstract

**Background:**

Immunotherapy is a promising cancer treatment, but its effectiveness is limited by the lack of specific strategies to target cancer stem cells (CSCs). CSCs a rare tumor cell subpopulation, are thought to drive tumor recurrence, metastasis, and therapy resistance and developing targeting CSCs is needed. This study investigates the efficacy of a CSC-based vaccine in preventing and treating breast tumor growth and metastasis in a mouse model.

**Methods:**

4T1-CSC was enriched by sphere formation and characterized by high expression of stemness genes using real-time PCR and higher in vivo tumorigenicity compared to parental cells. Whole CSC lysates were used as vaccine in prophylactic and therapeutic settings and their efficacy was compared with parental cells, normal saline and adjuvant-injected groups in terms of tumor growth, liver and lungs metastasis and survival. Additionally, the presence of antibodies against CSCs and parental cells was evaluated by flow cytometry and immunofluorescence staining in vaccinated mice.

**Results:**

CSC enrichment was confirmed by higher stemness gene expression and tumorigenicity in spheroid cells. CSC-Prophylactic vaccination significantly reduced tumor growth, lung and liver metastasis and improved survival compared to parental cell and control groups. Therapeutic vaccination reduced tumor growth until day 15, increases in survival and decreases in metastasis. Both vaccinations showed that CSC-vaccinated mice sera reacted more intensely with parental cells than with spheroid cells.

**Conclusion:**

CSC-based immunizations significantly reduce tumor growth and metastasis, enhancing survival. These findings underscore the potential of CSC-targeted therapies in cancer treatment, necessitating further research to develop effective breast cancer treatments.

**Supplementary Information:**

The online version contains supplementary material available at 10.1186/s13058-026-02242-7.

## **Introduction**

Breast cancer (BC) is the most commonly diagnosed cancer and the leading cause of cancer death in women worldwide [1] and metastasis accounts for the vast majority of breast cancer-related mortality [2]. In recent decades, significant progress has been made in breast cancer research, particularly in the areas of early detection, combination therapies, and immunotherapies. As a result, there has been a notable increase in the survival rates of patients diagnosed with this disease. Nevertheless, due to recurrence and metastasis, breast cancer continues to be the leading cause of cancer-related deaths among women on a global scale, highlighting the need for developing new therapeutic approaches [3].

Immunotherapy has been gaining attention as a new treatment for cancer and has proven its efficacy against malignancies over the past few years, making it an attractive strategy among the various available treatments [4]. Despite recent advances in cancer immunotherapy, the overall efficacy of these approaches is limited due to various reasons, including the lack of strategies to target cancer stem cells (CSCs). CSCs constitute a small proportion of the tumor cell population and possess the ability to self-renew and differentiation into heterogeneous tumor cells and play a central role in tumor recurrence, metastasis, and resistance to therapy [5–7]. In addition, some lines of evidence have shown that immune evasion may also be a characteristic of CSCs [8, 9]. Due to this, targeting and eradicating CSCs is the ideal approach for cancer treatment.

CSCs have been targeted by different strategies, including preventing their proliferation by inhibiting essential mitotic regulators [10], targeting signaling pathways involved in immune checkpoint blockade by therapeutic antibodies [11–13], and suppressing CSCs by oncolytic viruses [14]. Chimeric antigen receptor-modified T cells (CAR-T cells), which can specifically recognize antigens expressed on the cell surface of CSCs, is another way to target CSCs [15]. Stimulation of innate immune responses using dendritic cell (DC)-based vaccines [16, 17] and one of the current strategies to preferentially target CSCs is the use of whole CSC lysate as an antigen source to stimulate the immune system against CSCs [18]. Preclinical and clinical research on whole CSC-based vaccines is based on the concept that CSCs express distinctive neoantigens, as well as numerous tumor-associated antigens (TAA), which can potentially be a therapeutic target [17, 19, 20]. Although the main goal is to identify and characterize the CSC-specific antigens as a target for eradication of CSCs [17, 18].

Several studies have shown that whole CSC-based vaccines contain a broad spectrum of immunogens [21–23] that could stimulate immune responses; for example, CSC-lysate evokes in vitro and in vivo partial DC maturation and induces effective protective antitumor immunity [17]. In addition, mice vaccinated with CSC-lysate produce antibodies against colonosphere antigens [24]. From a functional view, several studies have shown that vaccination with CSC- lysate leads to a reduction in tumor volume [19, 23–26] and protects from experimental liver metastasis in colorectal cancer model [23]. Previous studies indicated that CSC-lysate vaccination leads to a reduction of CSCs in primary tumors and the recurrence rate of melanoma cancer [17, 22].

In the current study, we assessed the efficacy of prophylactic and therapeutic CSC-based vaccination using CSC-enriched cell lysate derived from the 4T1 cell line in the BALB/c mice model of breast cancer (4T1 tumor-bearing mice). The 4T1 cell line is highly tumorigenic and spontaneously metastasizes from the primary tumor to the liver and lung [27, 28] and mimics stage IV human breast cancer [29]. We assessed the feasibility and efficacy of CSC lysate as antigen sources to evaluate tumor growth and metastasis, survival rates, and humoral immune responses in an immunocompetent mouse model.

## Materials and methods

### Phase I: CSC enrichment using sphere formation culture and confirmation of cancer stem cell characteristics

#### 4T1 cell line culture

4T1 murine breast cancer cell line was purchased from the Cell Bank of Iran. The cells were cultured in Dulbecco-modified Eagle medium/F12 (DMEM/F12) (Gibco, Germany), supplemented with 10% heat-inactivated fetal bovine serum (FBS) (Gibco, Germany), and 100 U/mL penicillin and 100 µg/mL streptomycin (Biowest, France). The culture was maintained in a humidified incubator at 37 °C with 5% CO_2_. Cells were dissociated using 0.05% trypsin/EDTA solution (Gibco, Germany) and passaged when confluency reached 80–85%.

#### 4T1 CSC‑enriched spheroid culture

Spheroid formation assay was performed as described previously [24, 30]. In brief, 4T1 single cells (from second passages of two-dimensional culture) were cultured in poly-HEMA-coated dishes at a density of 5 × 10^4^ cells/mL in serum-free Dulbecco modified Eagle medium (DMEM/F12) (Gibco, Germany) supplemented with 10 ng/mL of basic fibroblast growth factor (bFGF) (PeproTech, USA) and 20 ng/mL of epidermal growth factor (EGF) (PeproTech, USA). During the cultivation, the morphology of the spheres was monitored by microscopic observation.

### Serial passages of 4T1 spheroids

To evaluate the capacity of spheres to form the next spheroid generation, spheres were collected after 3 days of cultivation. Spheres collection was performed by centrifugation for 1 min at 100 g and then supernatant was carefully removed. After washing the spheres with phosphate-buffered saline (PBS), cells were dissociated by trypsin-EDTA and gentle pipetting. The dissociated cells were cultured on a low attachment dish as mentioned above. 4T1 spheres were subjected to serial subculturing at three-day intervals for a total of five passages, and sphere morphology was investigated by microscopic observation during the five passages.

#### Scanning electron microscopy (SEM)

Spheres were washed twice with PBS and fixed in 2.5% glutaraldehyde (Sigma-Aldrich) for 20 min and then dehydrated with a series of increasing ethanol concentrations. After air drying at room temperature for 20 min, samples were coated with gold-palladium and subjected to analysis by a scanning electron microscope (SEM Seron Technology, AIS-2100, Korea).

#### mRNA expression analysis and real‑time qPCR

Total RNA was extracted from parental and spheroid cells (five sequential passages) utilizing the Hybrid-R Kit (GeneAll, Korea) according to the manufacturer’s instructions. The quantity and integrity of the RNA were evaluated using a NanoDrop spectrophotometer (ThermoFisher Scientific, USA) and agarose gel electrophoresis. Following DNaseI treatment, cDNA was synthesized with 1 µg of RNA using a cDNA synthesis kit (Yektatajhiz Azma, Iran). The real-time polymerase chain reaction (PCR) was performed using 1 µg of cDNA and PCR Master Mix Green-High Rox A325402-25 (Ampliqon, Denmark). The Rotor-Gene Q LightCycler (Qiagene, Germany) was utilized for this purpose.

The primer sets used for reverse transcription-quantitative polymerase chain reaction (RT-qPCR) analysis are listed in Table 1. The following thermal cycling conditions were used: initial denaturation step at 95 °C for 15 min, then 95 °C for 15 s, 60 °C for 15 s, and 72 °C for 15 s, for a total of 40 cycles. The relative expression of the Oct4, Sox2, and Nanog genes was measured relative to the expression of glyceraldehyde-3-phosphate dehydrogenase (Gapdh) as an internal control gene. The results were analyzed using the 2^−ΔΔCt^ relative quantification method.

**Table 1:** RT-qPCR primer sequences and PCR product sizes

Gene	Sequence	PCR product size (bp)
*Oct4*	F 5’- GTTCTCTTTGGAAAGGTGTTC − 3’ R 5’- GCATATCTCCTGAAGGTTCTC − 3’	147
*Nanog*	F 5’-TGATTTGGTTGGTGTCTTG − 3’ R 5’-TGTGATGGCGAGGGAAG − 3’	176
*Sox2*	F 5’- AAAGGGTTCTTGCTGGGTTT − 3’ R 5’- AGACCACGAAAACGGTCTTG − 3’	151
*Plac1*	F 5’- AGGAGAATCCTTCCTGGACG − 3’ R 5’- GTCGAGCACAGCACATTCAC − 3’	157
*Gapdh*	F 5’- AACTTTGGCATTGTGGAAGG − 3’ R 5’- CACATTGGGGGTAGGAACAC − 3’	222

#### Animals and ethical statement

Six- to eight-week-old female BALB/c mice were obtained from the Royan Institute (Tehran, Iran) and maintained under standard conditions at the animal laboratories of the Iran University of Medical Sciences (IUMS). All protocols have been approved by the IUMS animal research ethics committee (IR.IUMS.REC.1398.953). All animals were handled according to the committee guidelines during the study.

#### Evaluation of CSCs tumorgenicity potential compared to parental cells

To compare the tumorigenicity potential of 4T1 spheroid cells and parental cells, an equal number (10 × 10^3^ and 20 × 10^3^ cells in separate groups) of spheroid cells in the fifth passage (which had the highest stemness genes expression) and parental cells were injected subcutaneously into the opposite flank of the same BALB/c mice (*n* = 3 in each group). During the experimental period, tumor volumes were monitored and measured every other day. When the tumor grew to 18 mm in one dimension, the mice were euthanized.

#### Histological analysis of tumor tissues

Tumor tissues were removed and processed according to the standard protocol for paraffin section preparation [31]. The samples were fixed in a 10% buffered formalin solution. The paraffin-embedded tissues underwent sectioning at a thickness of 5 µm for hematoxylin and eosin (H&E) staining. The slides were evaluated by a microscope, and images were captured using a microscope camera (Nikon, Japan).

### Phase II: vaccination

#### Vaccine preparation

The 4T1 cell line and the 4T1 spheroid cells in the fifth passage were treated with 0.1 mM of 2-mercaptoethanol (2-ME) for 16 h before cell harvesting. Cells were collected and washed five times using PBS buffer. Protease Inhibitor Cocktail (Sigma, USA) was added to the collected cells, and cells were subjected to snap freeze-thaw for ten cycles alternating between − 80 and 37 °C, then centrifuged at 20,000 g for 20 min. The protein concentration of the supernatant was determined using the bicinchoninic acid (BCA) assay, according to the manufacturer’s instructions (TaKaRa Japan). For each immunization per mouse, 50 µg cell lysate was mixed with 25 µg of Poly (I: C) (Polyinosinic-Polycytidylic acid, Sigma, P1530) and 2 µg of CpG (Class B CpG oligonucleotide, ODN 1826) and injected into eight-week-old female BALB/c mice intraperitoneally (IP). In two control groups, normal saline and normal saline containing CpG-Poly (I: C) with the same concentration were used.

#### Prophylactic and therapeutic vaccination settings

Four groups of mice were considered in each set of prophylactic and therapeutic vaccinations. In each setting, the total number of female BALB/c mice was randomly divided into four groups. In the prophylactic setting, vaccination groups were immunized on days 0, 7, 14, and one week after the final vaccination, 10 × 10^4^ 4T1 cells in 0.1 mL of PBS were injected into the mammary glands of the mice using a 27-gauge needle. In the therapeutic setting, for tumor induction, 10 × 10^4^ 4T1 cells were injected subcutaneously into the mammary gland of the mice on day 0 in all four groups. Two vaccination groups were immunized on days 1, 8, and 15 intraperitoneally (IP) with CSC and parental cell lysates. In both prophylactic and therapeutic settings, two control groups received normal saline and CPG/poly IC.

#### Tumor size and survival evaluation

Tumor volumes were assessed every other day using a caliper. An experienced individual (S.S.) who was blinded to the study measured the tumor’s long and short diameters. The tumor volume was measured using the formula: tumor volume (mm3) = (length * width^2)/2. The mice’s survival rate was evaluated and recorded. The animals were euthanized after the tumors reached a size of 1.8 cm in their largest dimension.

#### Metastasis detection in lungs and liver of mice

##### Histological analysis of lungs and liver tissues

Tumors and organs of interest were fixed as mentioned above. The slides were screened for tumor metastases by a board-certified veterinary pathologist (H.G.).

##### Quantitative analysis of the metastases in lungs and liver

The Metastatic burden in the lung and liver was quantitatively assessed on hematoxylin and eosin (H&E)–stained tissue sections. For each experimental group, ten randomly selected microscopic fields per organ were analyzed at 10× magnification. Metastatic regions were manually delineated and their surface area was quantified using ImageJ software, in accordance with published methodology [32, 33]. The analysis specifically measured metastatic tumor cells area; consequently, associated stromal and immune cells area, such as neutrophils, were excluded from the measurements.

##### Quantification of Plac1 gene transcript as representative of 4T1 cells

We evaluated metastasis burden based on the level of Plac1 gene expression as a marker for detection of metastasis in 4T1 tumor-bearing mice [34]. Given that Plac1 as a cancer-testis antigen is expressed in the 4T1 cell line [35] and no expression was observed in normal mouse tissue except testis and placenta, we used the Plac1 molecule as representative of 4T1 cells. Mice were euthanized when one of the tumor dimensions reached 18 mm. Following this, their liver and lungs were immediately removed and kept at -80 degrees until use. Total RNAs were extracted from whole tissues using a one-step RNA reagent (Biobasic, Canada). The next steps were performed as indicated in secton “mRNA expression analysis and real‑time qPCR”.

The PCR program started with 15 min at 95 °C and was followed by 45 cycles (15 s at 95 °C, followed by 15 s at 60 °C, and 15 s at 72 °C). The results were analyzed using the 2^−ΔΔCt^ relative quantification method. Plac1 primer sequences are shown in Table 1. Gapdh was used as an internal control.

### Phase III: assessment of antibody production against CSC and parental cell antigens in immunized mice

In order to assess the antibody production against 4T1 and 4T1-CSCs antigens in all groups, the serum of immunized mice was collected, and in each group, equal volumes of serum from each mouse were pooled and used in subsequent investigations.

#### Flow cytometry

Cell suspensions with viability higher than 95% were used for flow cytometry analysis. Cells were washed twice with cold PBS. Then, nonspecific bindings were blocked by 5% normal sheep serum along with 2% FBS in PBS for 30 min on ice. After washing, parental cells and spheroids were incubated with a 1:50 dilution of pooled mouse sera from each vaccinated group for 45 min at 4 °C. The same dilution of non-immune mice sera was used as an isotype control. Four washing steps were performed with PBS containing 2% FBS. FITC-conjugated sheep anti-mouse IgG (Avicenna Research Institute (ARI), Tehran, Iran), with a dilution of 1:100, was applied for 30 min. In addition to isotype control, two other controls were also used. Untreated cells for evaluation of cell-intrinsic autofluorescence and the cells that received PBS instead of mice sera. Data acquisition was conducted using an Attune NxT flow cytometer (Thermo Fisher Scientific, USA), and data analysis was performed using FlowJo software VX.

#### Immunofluorescence staining (IF)

Parental cells and spheroid cells were fixated with 4% paraformaldehyde for 30 min at room temperature (RT), permeabilized with 0.2% Triton X-100, and washed twice with PBS containing 2.5% BSA. Then, 5% normal sheep serum and 2.5% BSA in PBS were used to block nonspecific binding for 30 min at room temperature. Subsequently, three more washing steps were carried out with a washing buffer. Following this, 4T1 parental cells and spheroids were incubated for 90 min with a 1:50 dilution of mice pool sera from each vaccinated group. Serum from non-immunized mice at a similar dilution was used as isotype control. Consequently, FITC-conjugated sheep anti-mouse IgG (Avicenna Research Institute (ARI), Tehran, Iran) with a dilution of 1:100 was applied at room temperature for 45 min. Following the washing, 1 µg/mL DAPI solution (4′, 6-diamidino-2-phenylindole, Sigma-Aldrich, Germany) was added to stain the nuclei, and cells were evaluated by fluorescence microscopy (Olympus IX71; outfitted with a 460 nm filter for DAPI and a 510 nm filter for FITC). Negative controls were the same in section “Flow cytometry”.

#### Measurement of anti-Plac1 antibody in the sera of mice vaccinated with CSC lysate, parental cell lysate and normal saline injected groups

To quantify anti-Plac1 antibody levels across all groups in both prophylactic and therapeutic settings, serum was collected from immunized mice. For each group, serum from individual mice was pooled in equal volumes and subjected to an enzyme-linked immunosorbent assay (ELISA). The mouse recombinant Plac1 and monoclonal antibodies against mouse Plac1 (clone: 5B11) were generous gifts from Professor Amir-Hassan Zarnani. For performing ELISA assay, mouse recombinant Plac1 [36] was coated onto plates at a concentration of 10 µg/mL in PBS (pH 7.4) for 30 min at 37 °C, followed by an overnight incubation at 4 °C. The plates were then washed three times with PBS containing 0.05% Tween-20 (PBS-T). Non-specific binding sites were blocked with 2% bovine serum albumin (BSA) in PBS-T for 90 min at 37 °C, after which the plates were washed three additional times with PBS-T. For the standard curve, an anti- Plac1 monoclonal antibody (clone: 5B11) [36] was serially diluted in eight concentrations, ranging from 2 µg/mL to 0.015 µg/mL. Pooled sera from each experimental group were diluted 1:100 in diluent, added to the plates, and incubated for 90 min at 37 °C. Unbound components were removed by performing three subsequent washes with PBS-T. Then, horseradish peroxidase (HRP)-conjugated rabbit anti-mouse immunoglobulin (Ig) antibody was added and incubated for 60 min. The plates were washed again, followed by the addition of tetramethylbenzidine (TMB) substrate. The enzymatic reaction was stopped after 15 min by adding 15 µL of 20% sulfuric acid (H_2_SO_4_). Finally, the absorbance was measured at 450 nm using a microplate reader (Synergy HTX, BioTek, USA). For measurement of anti-Plac1 antibody in mice sera, the mean absorbance for each standard was plotted against its corresponding concentration. The concentration of anti-Plac1 antibody in mice sera was subsequently determined by extrapolating their mean OD values from the generated standard curve. All samples were assayed in duplicate, and any values falling below the range of the standard curve were considered non-quantifiable.

### Statistical analysis

GraphPad Prism, version 8.0 (GraphPad Software, La Jolla, CA, USA, www.graphpad.com), was utilized to analyze the data. Results were evaluated using the student’s t-test. At *P* < 0.05, differences were considered to be statistically significant. The Mann-Whitney U test, a two-tailed, nonparametric probability test, was used to compare the volumes of tumors in vaccinated and control mice. The log-rank (Mantel-Cox) test was used to evaluate survival. A one-way ANOVA analysis was performed to compare the level of expression of the stemness gene in subsequent passages.

## Results

### Phase I: enrichment of CSCs using sphere formation culture and confirmation of stem cell characteristics

#### Enrichment of 4T1 cancer stem cells using spheroid formation assay

**Fig. 1:**
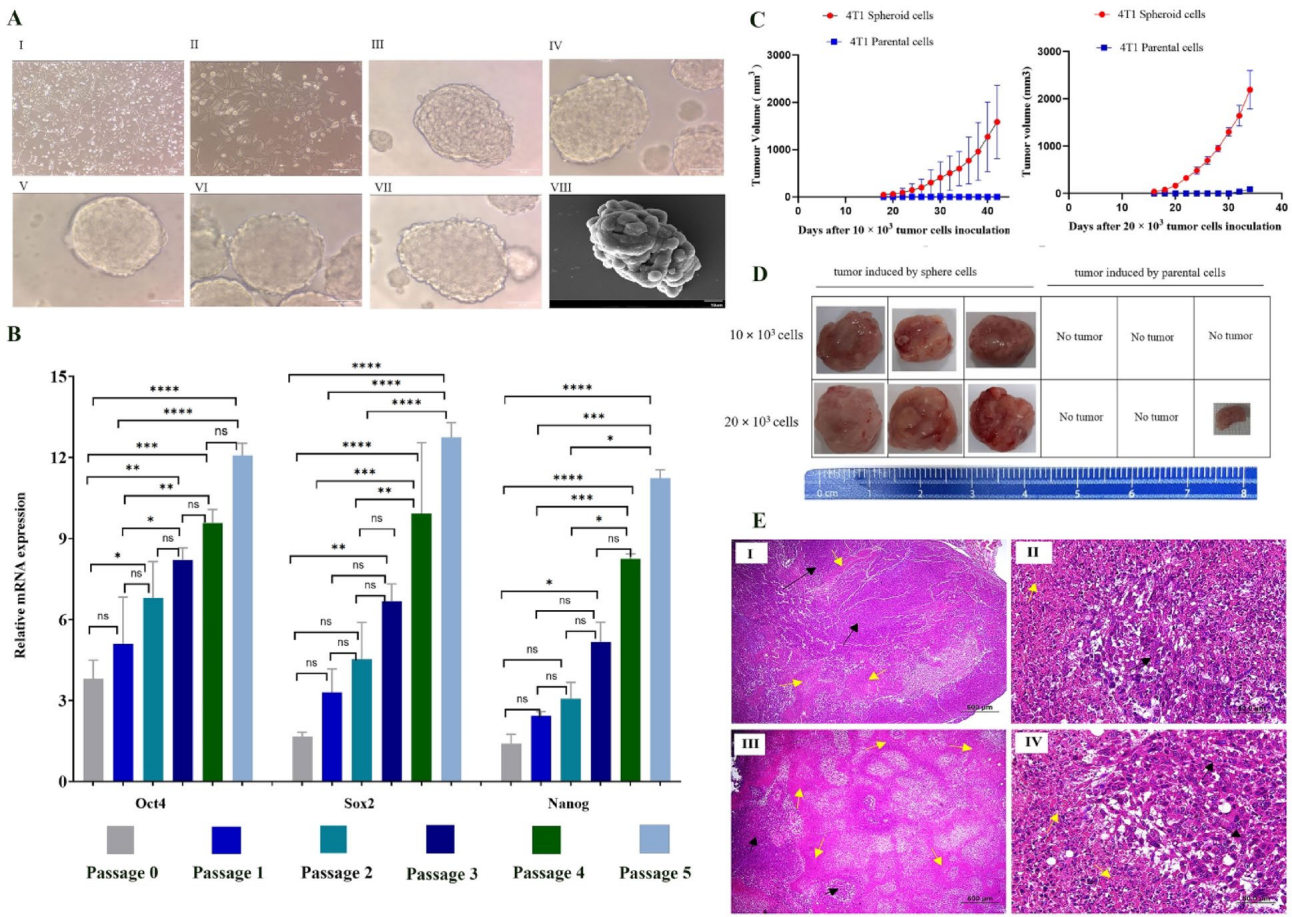
**A** 4T1 parental cells and spheroid cells morphology in culture media. I: 4T1 parental cells in adherent culture with 100x magnification. II: 4T1 parental cells in adherent culture with 400x magnification. III, IV, V, VI, VII: spheroid cells derived from 4T1 cell line in non-adherent culture in passage 1–5 with 400x magnification. VIII: SEM image of a 4T1 spheroid. **B** A comparative analysis of the expression levels of the stemness genes *Oct4*, *Sox2*, and *Nanog* in parental cells and CSCs in five consecutive passages. As shown in the diagram, the expression levels of the stemness genes were highest at passage five compared to earlier passages of spheroid cells. **C** Comparative tumorigenic capacity of 4T1 spheroid cells and their parental cells. An equal number of 4T1-spheroid and 4T1-parental cells were injected into the opposing side of the mouse, 20 × 10^3^ cells (*n* = 3), 10 × 10^3^ cells (*n* = 3). **D**: tumor induced in both groups (10 × 10^3^ cells and 20 × 10^3^ cells) using sphere cells, no tumors were observed by injecting the 10 × 10^3^ parental cells, and only one tumor was induced in the group of 20 × 10^3^ cells. **E** Histopathological evaluation of tumor tissues using H&E staining. I & II: Tumor tissue generated by 4T1 parental cells. I: Infiltration of ovoid to round neoplastic cells with solid pattern (black arrow) and foci of necrosis (yellow arrow) were shown. II: Higher magnification of slide I. Tumor cells with large vesicular nuclei and multiple prominent nucleoli (black arrow) and necrotic foci (yellow arrow) were seen. III & IV: Tumor tissue generated by spheroid cells. III: The neoplastic cells in solid pattern (black arrow) and multiple foci of necrosis (yellow arrow) were observed. IV: Higher magnification of slide III (H & E staining, Scale bar: A & C: 500 μm, B & D: 50 μm)

To enrich CSCs, adherent 4T1 cells in the second passage (Fig. 1A I, II) were transferred to serum-free and non-attachment cultures. Compact and stable spheroids with a round shape structure were formed after 3 days. The morphology of five consecutive passages of 4T1 spheroid is shown in Fig. 1A III, IV, V, VI, VII. SEM imaging showed the compact structure and cell surface connections of the 4T1-sphere (Fig. 1A VIII).

#### High transcript expression levels of Oct4, Sox2, and Nanog in spheroid cells compared to parental cells

Transcript expression analysis of Oct4, Sox2, and Nanog as master regulators of pluripotency and self-renewal in CSCs showed that the expression level of these stemness genes was significantly higher in spheroids-derived cells compared to parental cells. In addition, the stemness expression levels were significantly increased in a stepwise manner from passage zero to passage five in sphere cells (Fig. 1B).

#### High tumorigenic potential of spheroid cells compared to parental cells

To assess the tumorigenic potential of the 4T1-derived spheroids in comparison to 4T1 parental cells, an equal number of spheroid cells in the fifth passage and parental cells were subcutaneously injected into opposite sides of the Balb/c mouse. Two cell numbers (10 × 10^3^ cells and 20 × 10^3^ cells) were used for tumor induction in separate groups of mice. Tumor volume analysis showed that, while tumors were induced in both groups (10 × 10^3^ cells and 20 × 10^3^ cells) using sphere cells, no tumors were observed by injecting the 10 × 10^3^ parental cells, and only one tumor was induced by injecting the 20 × 10^3^ parental cells, indicating that sphere cells possess more tumorigenic potential compared to parental cells. In 10 × 10^3^ and 20 × 10^3^ sphere cells groups, tumors were palpable on average on days 18 and 16, respectively. As expected, tumor growth was slower in the 10 × 10^3^ cells group than in the other group. The mean tumor volumes on day 30 in 10 × 10^3^ and 20 × 10^3^ sphere cells groups were 409 mm^3^ and 1304 mm^3^, respectively (Fig. 1F, G, H). In the 10 × 10^3^ cells group, one mouse was sacrificed on day 42, and two mice were sacrificed on day 50 after tumor induction. In the 20 × 10^3^ cells group, all three mice were sacrificed on day 34 after tumor induction. Based on the above findings, sphere cells were considered a CSC-enriched population for further investigation.

#### Histological analysis of tumor tissues

Histopathological analysis showed that the tumor tissue consisted of sheaths of ovoid to round cells with poorly defined cytoplasm and often with vesiculated and prominent nucleoli in the only tumor that was induced by injection of 20 × 10^3^ parental cells. The neoplastic cells appeared in a solid pattern, and there were some foci of necrosis (Fig. 1E-I, II). In tumors generated by spheroid cells, the tumor cells had large hyperchromatic or large vesicular nuclei, single and/or multiple prominent nucleoli, and a relatively small amount of cytoplasm. There was a significant sign of intra-tumor hemorrhage and necrosis. Moreover, invasion of tumor cells into normal adjacent parenchyma and vascular structure was also observed (Fig. 1E-III, IV).

### Phase II: effects of therapeutic and prophylactic CSC lysate vaccination on tumorigenesis, metastasis, and survival in breast cancer model

**Fig. 2:**
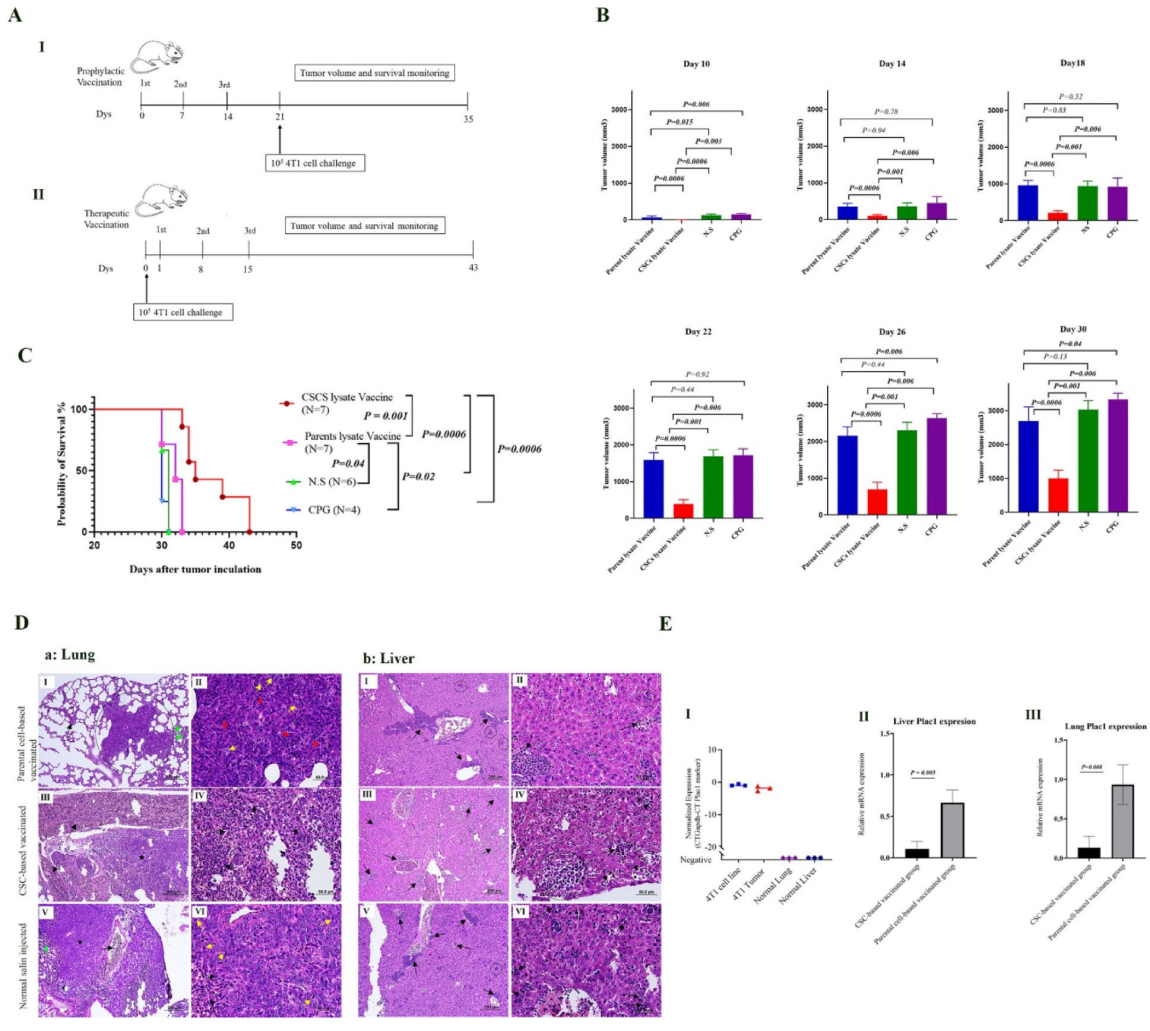
Prophylactic CSC-based vaccination reduces tumorigenesis, metastasis and increase survival of 4T1-tumor bearing mice. **A** Prophylactic and therapeutic vaccination schemes. I: In the prophylactic setting, vaccination groups were immunized on days 0, 7, and 14. On day 21, 10 × 10^4^ 4T1 cells were injected for tumor induction. II: In the therapeutic setting, for tumor induction, 10 × 10^4^ 4T1 cells were injected on day 0 for tumor induction, and mice were immunized on days 1, 8, and 15. **B** Effect of prophylactic CSC-based vaccination on tumor growth. Tumor growth comparative analysis was performed following tumor induction in vaccinated mice. The group that received the CSC-based vaccine exhibited a significantly reduced rate of tumor growth on all days in comparison to the other groups. **C** The Kaplan-Meier analysis of prophylactic-based vaccinated mice survival. The survival time of mice immunized with CSC-based vaccine was significantly longer than that of mice immunized with parental cell-based vaccine (*P* = 0.001) and exhibited a considerably higher rate of survival in comparison to the normal saline and CPG control groups (*P* = 0.0006 and *P* = 0.0006). **D** Histopathological analysis of lungs and liver of vaccinated mice. Part a: Lungs. I: parental cell-based vaccinated group: metastatic foci (circle), diffuse infiltration of inflammatory cells in alveolar walls (black arrow), and also alveolar atelectasis (green arrow) were observed. II: Higher magnification of slide I, nuclear polymorphism (red arrow), and neutrophilic infiltration (yellow arrow) within the metastatic nodule were detected. III: CSC-based vaccinated group: metastatic nodule (star) and active phase of inflammatory cell migration from the vascular system (black arrow) have been indicated. IV: Higher magnification of slide III; the noticeable amount of vesiculated nuclei with prominent nucleoli of tumor cells (star) and also the infiltration of neutrophils (black arrow) within the metastatic nodule were observed. V: Normal saline-injected group: metastatic foci (star), collapse of pulmonary parenchyma (green arrow) adjacent to metastatic foci, and active phase of neutrophilic infiltration from the vascular system (black arrow) have been shown. VI: Higher magnification of slide V; presence of numerous scattered mitotic divisions (yellow arrow); and also, granulocyte infiltration (black arrow) within metastatic foci were seen. Part b: Liver. I: Parental cell-based vaccinated group: hematopoietic centers near blood vessels (black arrow) and also metastatic foci in the parenchyma (circle) were observed. II: Higher magnification of slide II. III: In the CSC-lysate-vaccinated group, numerous extramedullary hematopoietic foci are detected (black). IV: Higher magnification of slide III; also metastatic nodule (circle) in the parenchyma were seen. V: Normal saline-injected group: Some foci of breast cancer metastases (circle) were detected in the liver as well as extramedullary hematopoietic foci (black arrow). VI: Higher magnification of slide V (H & E staining, scale bar: I, III, V: 200 μm and II, IV, VI: 50 μm). **E** Prophylactic CSC-based vaccination significantly reduces metastatic burden in the lungs and liver of 4T1-tumor bearing mice. I: Plac1 expression was detected in 4T1 and 4T1-Tumor. No Plac1expression was detected in normal lungs and liver of normal mice. II: The expression level of the Plac1 was detected in whole liver of prophylactic immunized tumor-bearing mice and the result showed Plac1 expression level in parental cell-based vaccinated group was significantly higher than CSC-based vaccinated group (*P* = 0.005). III: The expression level of the Plac1was detected in whole lungs of prophylactic immunized tumor-bearing mice and the result showed Plac1 expression level in parental cell-based vaccinated group was significantly higher than CSC-based vaccinated group (*P* = 0.008)

Fifty (50) µg of the prepared cell lysate, along with CpG (3 µg) and Poly I: C (25 µg) as adjuvants, were used as a vaccine for each mouse in both prophylactic and therapeutic vaccination groups. The vaccination schema is illustrated in Fig. 2A I, and II.

#### Prophylactic CSC-based vaccination effects

##### Prophylactic CSC-based vaccination reduces tumor growth and increases survival

To determine the tumor growth rate over time, tumor volume was measured every other day, and comparative analysis was performed and continued until the 30th day after tumor induction, the day when the largest dimension of a tumor in each of the groups reached 18 mm. As shown in Fig. 2B, CSC-based prophylactic vaccination significantly reduced tumor growth in comparison to parental cell-based vaccination and also in comparison to control groups (Fig. 2B). The comparative analysis of tumor size was assessed as long as all mice in all four groups were alive.

In addition, parental cell-based vaccination led to a significant reduction in tumor volume compared to the normal saline-injected group until the 12th day (*P* = 0.001). However, from the 14th day onwards, no significant difference was observed between these two groups (Fig. 2B).

Survival analysis by log-rank and Mantel-Cox test revealed that survival of the CSC-based vaccinated group was significantly higher than the parental cell-based vaccinated group (*P* = 0.001) and two other groups (*P* = 0.0006 and *P* = 0.0006) (Fig. 2C). Also, the survival of the parental cell-based vaccinated group was significantly higher than the normal saline-injected group (*P* = 0.04). The mean survival times for the CSC-based vaccinated group, the parental cell-based vaccinated group, normal saline, and CPG/Poly I: C injected groups were 37.2, 31.8, 30.6, and 30.2 days, respectively.

##### Histopathological analysis of lung and liver of vaccinated tumor-bearing mice

Histopathological analysis of the two main metastatic organs (lungs and liver) was performed in the prophylactic vaccinated mice group. In the CSC-based vaccinated group, some metastatic nodules, inflammatory cell infiltration, and hemorrhage were detected in the lung. In the parental cell-based vaccinated group, lung metastases consisted of small nodules with round to spindle-shaped cells with little cytoplasm. An active phase of inflammatory cell migration from the vasculature was also observed. In the lung of normal saline-injected groups, metastatic nodules were observed, and in each nodule, tumor cells were characterized by large hyperchromatic nuclei and a small amount of cytoplasm. The alveolar walls showed diffuse and intense inflammatory infiltration in the neighborhood of metastasis foci and even within them. In the lung of CSC-based vaccinated group, lower metastatic nodules, and mitotic index were detected in comparison to the parental cell-based vaccinated group and normal saline-injected group. Higher inflammation and mitotic index and large hyperchromatic nuclei were observed in lungs of normal saline-injected group compared to other groups (Fig. 2D a).

In the liver of the CSC-based vaccinated group, numerous extramedullary hematopoietic centers were observed near blood vessels and within the parenchyma and also some metastatic foci were identified in the liver parenchyma. In the parental cell-based vaccinated group, metastatic foci were detected in liver parenchyma. A large number of granulocytes were observed in the blood vessels and liver sinusoids. Also, islands of extramedullary hematopoiesis were found near blood vessel walls. In the normal saline-injected control group, a significant number of extramedullary hematopoietic foci were seen in the liver. The liver parenchyma displayed small foci of breast cancer metastasis. In the liver of the CSC-based vaccinated group, inflammation and metastatic nodules were lower than in the parental cell-based vaccinated group and the normal saline-injected group (Fig. 2D b).

##### Prophylactic CSC-based vaccination decreases metastasis burden in the liver and lungs in tumor-bearing mice

To evaluate the efficacy of the vaccination strategies, the metastatic burden in the lungs and liver was quantified across all experimental groups. To assess metastatic burden, the percentage of parenchymal area occupied by metastases in lung and liver tissues from 4T1 tumor-bearing mice was estimated quantitatively using ImageJ software on hematoxylin and eosin (H&E)-stained sections.

Analysis of metastases area in the lungs and livers of tumor-bearing BALB/c mice revealed a reduction in the percentage of metastases area in the CSC group compared to the other groups (Fig S1 A). In the prophylactic setting, the mean percentage of metastasized tissue in the lung of CSC-vaccinated group was 4.68% compared to 13.8% in parental- cell vaccinated group (not significant). Comparison of metastasis area in the liver showed a reduction in metastasis in CSC-vaccinated group compared to parental cell-vaccinated group (1.28% vs. 4.0%, *p* < 0.01). Metastasis burden in lungs and liver of the normal saline injected group were 21.2% and 4.94%, respectively.

In addition to evaluate metastasis burden in prophylactic CSC-based vaccinated 4T1-tumor bearing mice compared to other groups, the expression level of the Plac1 gene, a cancer-testis antigen with no expression in normal mouse tissues and ectopic expression in the 4T1 cell line, and the 4T1 tumor (Fig. 2E -I) was assessed in the whole liver and lungs of mice. The Plac1 transcript expression level analysis revealed that the CSC-based vaccinated group had significantly lower levels of Plac1 expression in the whole liver and lungs compared to the group vaccinated with parental cell lysate (*P* = 0.005and *P* = 0.008, respectively) (Fig. 2E-II, III). These results indicate that prophylactic CSC-based vaccination decreases the burden of metastasis in the liver and lungs in tumor-bearing mice.

#### Therapeutic CSC-based vaccination effects

##### Therapeutic CSC-based vaccination reduces tumor growth and increases survival compared to control groups

**Fig. 3:**
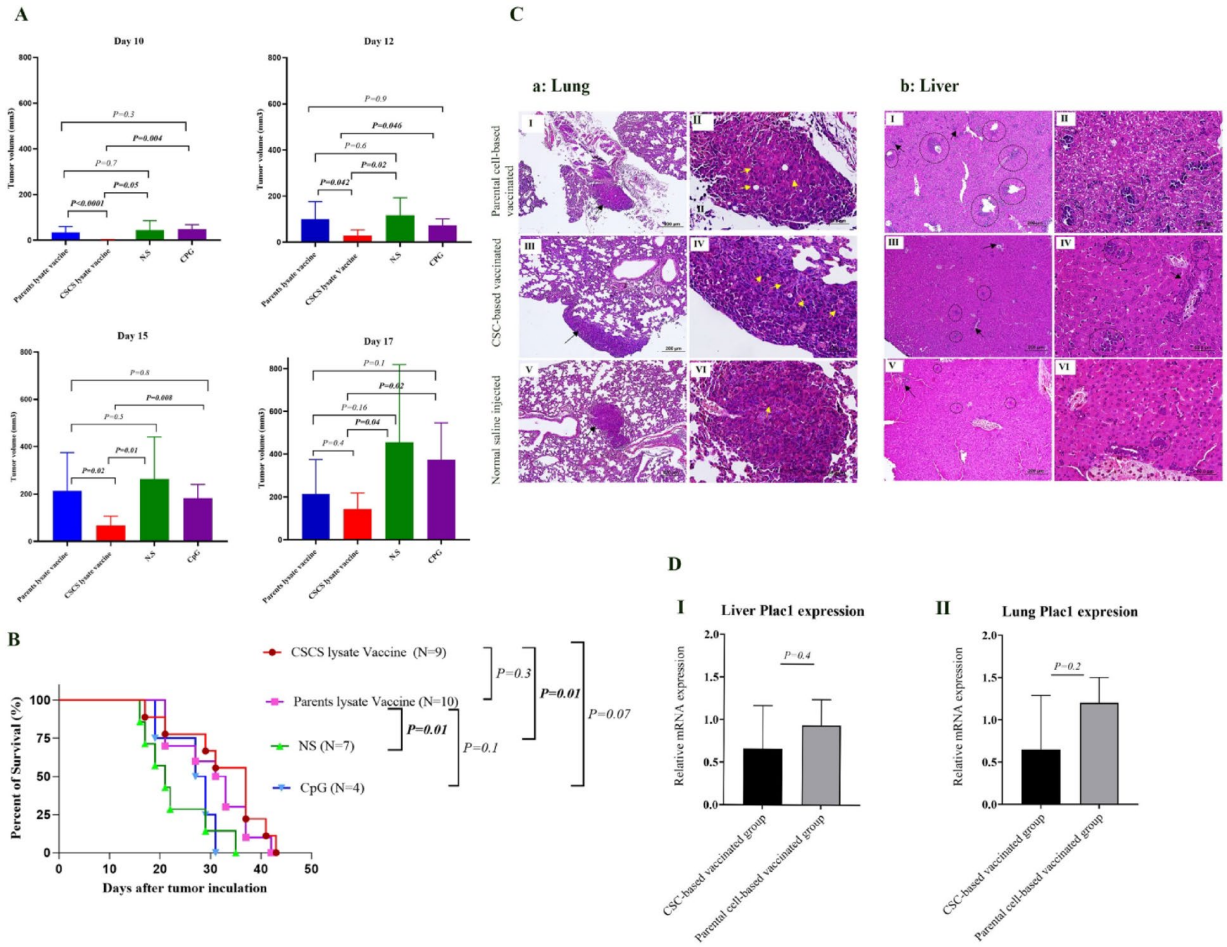
Therapeutic CSC-based vaccination reduces tumorigenesis and metastasis and increases survival of 4T1-tumor bearing mice. **A** Effect of therapeutic CSC-based vaccination on tumor growth. The CSC-based vaccinated group exhibited a significantly reduced rate of tumor growth until days 15, in comparison to the other groups. **B** The Kaplan-Meier analysis of therapeutic-based vaccinated mice survival. The survival time of CSC-based vaccinated mice was longer than of parental cell-based vaccinated mice, but it was not statistically significant (*P* = 0.3). There was a significant increase in survival rates in CSC-based vaccinated group in comparison to the normal saline-injected group (*P* = 0.01) and also, there was a significant increase in survival in parental cell-based vaccinated group, in comparison to the normal saline-injected group (*P* = 0.03). **C** Histopathological analysis of lungs and liver of vaccinated mice. Part a: Lungs. I: parental-based vaccinated group: metastatic foci (black arrow) were detected. II: higher magnification of slide I; mitotic divisions (yellow arrow) were detected. III: CSC-based vaccinated group: metastatic nodule (black arrow) was observed. IV: higher magnification of slide III, mitotic cells (yellow arrow) were observed. V: normal saline-injected group: some foci of breast cancer metastasis (circle) and extramedullary hematopoiesis were seen (black arrow). VI: higher magnification of slide V; mitotic division (yellow arrow) were seen. Part b: Liver. I: parental cell-based vaccinated group: extramedullary hematopoietic were observed near blood vessels (black arrow) and also metastatic foci in parenchyma (circle) were detected. II: higher magnification of slide I. III: CSC-based vaccinated group: metastatic nodules were detected in parenchyma (circle) and also extramedullary hematopoietic (black arrow) were observed. IV: Higher magnification of slide III. V: normal saline-injected group: some foci of breast cancer metastasis (circle) and extramedullary hematopoiesis (black arrow) were detected. VI: Higher magnification of slide V (H & E, scale bar: I, III, V: 200 μm and II, IV, VI: 50 μm). **D** Therapeutic CSC-based vaccination reduced the metastatic burden in the lungs and liver of 4T1 tumor bearing mice. I: The expression level of the Plac1 in the whole liver of therapeutically immunized tumor-bearing mice showed that Plac1 expression in CSC-based vaccinated group was lower than parental-based vaccinated group but this decrease was not significant (*P* = 0.4). II: The expression level of the Plac1 in the whole lungs of therapeutically immunized tumor-bearing mice showed that Plac1 expression in CSC-based vaccinated group was lower than parental-based vaccinated group but this decrease was not significant (*P* = 0.2)

In order to examine therapeutic vaccination efficacy on tumor progression, tumor volume was measured every other day, and comparative analysis was performed. Tumor volume analysis revealed reduced tumor growth in the CSC-based vaccinated group in comparison to the parental cell-based vaccinated group on days 10, 12, and 15 after tumor induction (*P* < 0.0001, *P* = 0.042, and *P* = 0.02, respectively). Although, after the day 17 of tumor induction, there was still a decrease in tumor growth in the CSC-based vaccination group compared to parental-based vaccination, this difference was not statistically significant (*P* = 0.4) (Fig. 3A).

Survival analysis in the therapeutic vaccination group indicated no significant differences in survival for the CSC-based vaccinated group compared to the parental cell-based vaccinated group (*P* = 0.3). The CSC-based vaccinated group showed a significant increase in survival compared with the normal saline-injected group (*P* = 0.01). Additionally, a statistically significant difference was observed in survival rates between mice vaccinated with parental cell lysate and those receiving normal saline (*P* = 0.03). The mean survival times for the CSC-based vaccinated group, the parental cells-based vaccinated group, the normal saline-injected groups, and the CPG/Poly I: C-injected group were 32.5, 30.5, 22.7, and 26.5 days, respectively (Fig. 3B).

##### Histopathological analysis of the lungs and liver of vaccinated tumor-bearing mice

Histological analysis was performed on the lung and liver of mice receiving the therapeutic vaccine. In the CSC-based vaccinated group, small metastatic lesions were detected in the lung. Tumor cells exhibited hyperchromatic nuclei and scant cytoplasm. Interstitial granulocytic infiltration of the alveolar wall without alveolar involvement was also observed. In the parental cell-based vaccinated group, some active metastatic foci were detected in the pulmonary parenchyma. The tumor cells were polyhedral to spindle-shaped with pleomorphic nuclei. Increased levels of infiltrated neutrophils in the alveolar walls were also observed. In the normal saline-injected group, small metastatic lesions were observed. Tumor cells had large hyperchromatic or large vesicular nuclei and single and/or multiple prominent nucleoli. Moreover, interstitial granulocytic infiltration and foamy macrophages in the pulmonary parenchyma were seen (Fig. 3C a). In the livers of the three groups, there were many small foci of breast cancer metastasis and also extramedullary hematopoiesis. Also, diffuse inflammatory infiltration was observed (Fig. 3C b). In the lungs and liver of the CSC-based vaccinated group, metastatic nodules, and inflammations were lower than the parental cell-based vaccinated group and normal saline-injected group.

##### Therapeutic CSC-based vaccination decreases the metastasis burden in the liver and lungs in tumor-bearing mice in a non-statistically significant way

Metastatic burden was evaluated by calculating the proportion of lung and liver parenchyma affected by metastatic foci in 4T1 tumor–bearing BALB/c mice. Quantification was performed on hematoxylin and eosin (H&E)–stained sections using ImageJ software. This analysis showed that the metastatic area was lower in the CSC-vaccinated group compared with the other groups (Fig S1 B). The mean metastatic burden in the CSC-vaccinated group was 13.41% in the lung and 2.48% in the liver, compared with 21.64% and 3.72% in the parental vaccine group (not significant), and 27.47% and 5.6% in the normal saline injected group, respectively.

Moreover, Metastasis burden was assessed in vaccinated mice by measuring the Plac1 transcript expression level. The results revealed that the CSC-based vaccinated group had a decreased Plac1 transcript expression level in the liver and lungs compared to the parental cell-based group; however, the difference was not statistically significant (*P* = 0.4 and *P* = 0.2 respectively) (Fig. 3D I, II).

#### Assessment of humoral responses against 4T1-CSCs and 4T1-parental cell antigens in tumor-bearing mice following prophylactic and therapeutic vaccination using flow cytometry, immunofluorescence staining and ELISA

To assess anti-CSC and anti-parental antibodies in prophylactic and therapeutically vaccinated mice, sera were collected, and the immunoreactivity of the sera against CSC and parental cells was investigated.

##### Flow cytometry

**Fig. 4:**
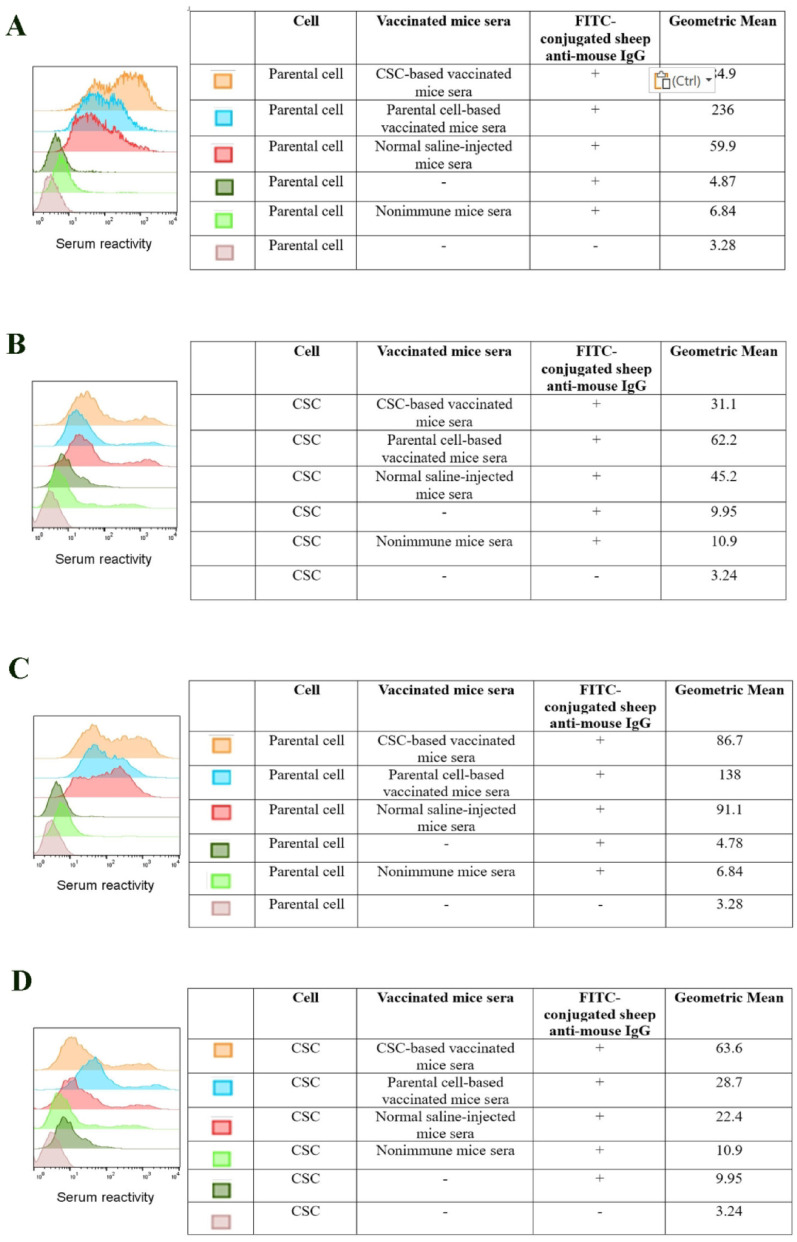
**A**, **B** Flow cytometry analysis of vaccinated mice sera immunoreactivity with 4T1 spheroid and parental cell populations in prophylactic vaccination group. Both pooled serum from CSC-based vaccinated mice and pooled serum from parental cell-based vaccinated mice reacted to the parental cells with stronger MFI compared to CSCs. Isotype control used to confirm the immunoreactivity specificity. **C**, **D** Flow cytometry analysis of vaccinated mice sera immunoreactivity with 4T1 spheroid and parental cell populations in therapeutic vaccination group. Parental cells exhibited a greater MFI (86.7) than spheroid cells (63.6) when serum from CSC-based vaccinated mice was used. Additionally, parental cells showed a higher MFI (138) than spheroid cells (28.7) when parental cell-based vaccinated mice serum was used

Flow cytometry analysis, based on the percentage of stained cells, showed that nearly all of the CSC and parental cells were stained by CSC and parental vaccinated mice sera (data not shown). Consequently, the flow cytometry analysis was performed using mean fluorescence intensity (MFI). The immunoreactivity of immunized mice sera (pooled sera) against parental cells and CSCs is shown in Fig. 4A, B, C, and D.

In prophylactic vaccination, the results of flow cytometry analysis revealed that the MFI of parental cells was stronger (84.9) compared to CSCs (31.1), when serum from CSC-based vaccinated mice was used. Also, the use of pooled serum from parental cell-based vaccinated mice showed the MFI of parental cells was stronger (236) in comparison to spheroid cells (62.2). Furthermore, pooled serum from normal saline-injected mice showed reactivity towards both CSCs and parent cells (MFI = 45.2 and 59.9, respectively) (Fig. 4A, B).

Flow cytometry analysis in the therapeutic vaccination demonstrated that pooled serum from CSC-based vaccinated mice reacted stronger with parental cells (MFI = 86.7) compared to CSCs (MFI = 63.6), as well as when pooled serum from parental cell-based vaccinated mice was used, the MFI of parent cells was stronger (138) than spheroid cells (28.7). In addition, the serum of mice injected with normal saline reacted with CSC (22.4) and parental cells (91.1) (Fig. 4C, D).

##### Immunofluorescence staining

**Fig. 5:**
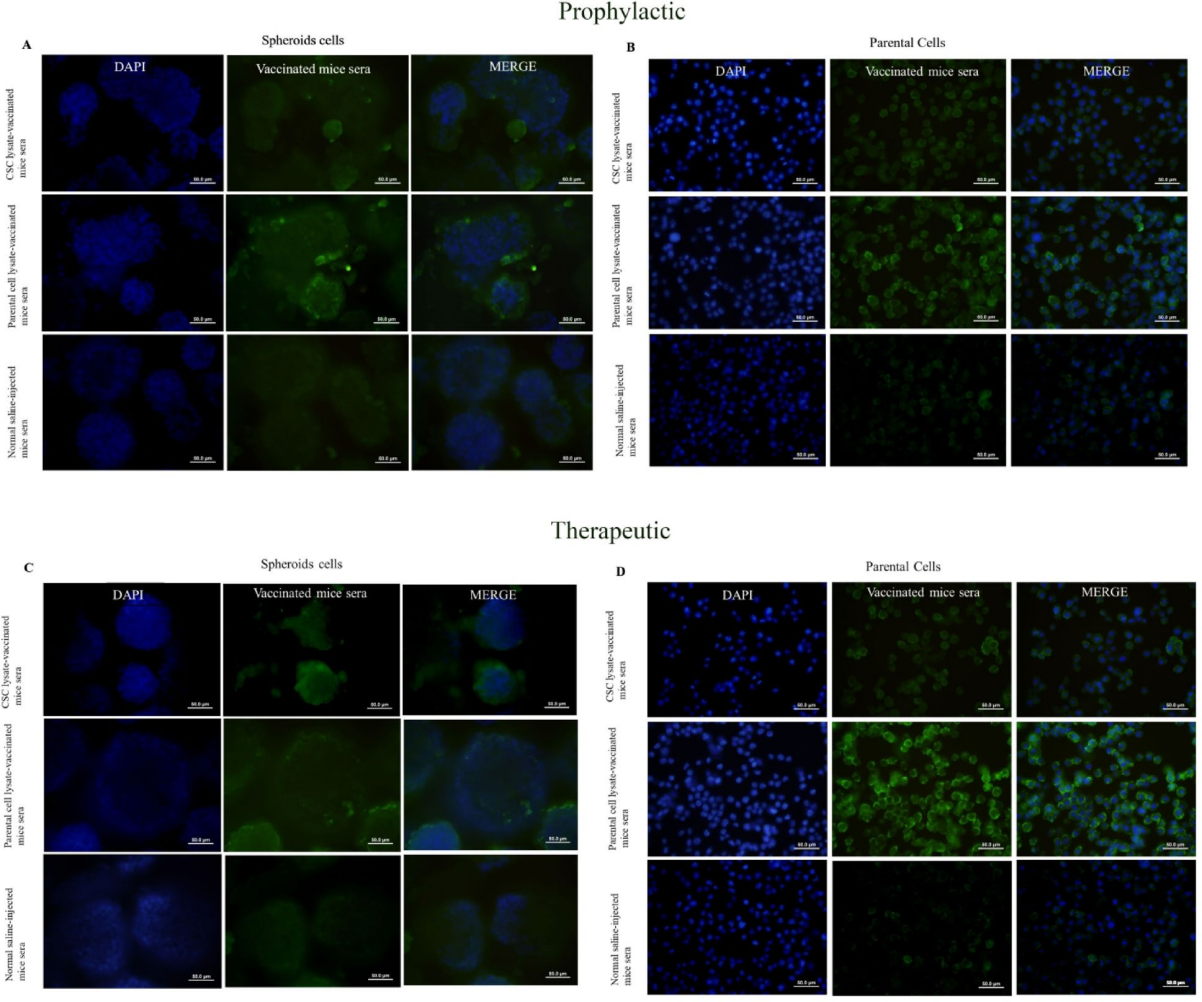
**A**, **B** Prophylactic immunization with CSC lysate and parental lysate induced the production of antibodies against spheroid and parental cell antigens. **A** 4T1 spheroids were incubated with CSC and parental cell, and normal saline vaccinated mice sera. Serum of parental-vaccinated sera showed more reactivity against 4T1 spheres as compared to CSC-based vaccinated mice sera. **B** 4T1-parental cells were incubated with CSC and parental cell, and normal saline vaccinated mice sera. Serum of parental-vaccinated sera showed more reactivity against 4T1-parental cells as compared to CSC-based vaccinated mice sera. **C**, **D** Therapeutic immunization with CSC lysate and parental lysate induced the production of antibodies against spheroid and parental cell antigens. **C** 4T1 spheroids were incubated with CSC and parental cell, and normal saline vaccinated mice sera. Serum of parental-vaccinated sera showed more reactivity against 4T1 spheres as compared to CSC-based vaccinated mice sera. **D** 4T1-parental cells were incubated with CSC and parental cell, and normal saline vaccinated mice sera. Serum of parental-vaccinated sera showed more reactivity against 4T1-parental cells as compared to CSC-based vaccinated mice sera

As shown in Fig. 5A, B, C, and D, immunofluorescence staining exhibited that in both prophylactic and therapeutic experiments, pooled sera from CSC-based vaccinated mice reacted to parental cells more intensively relative to CSC cells, similar to flow cytometry results. Also, pooled sera from parental cell-based vaccinated mice reacted to parental cells with more intensity compared to CSC cells. Additionally, CSCs and parental cells were recognized by pooled sera from normal saline-injected mice.

##### Quantification of anti-Plac1 antibody levels by ELISA

In the assessment of anti-Plac1 antibody levels following prophylactic vaccination, significant differences were observed between experimental groups. Mice vaccinated with CSC lysates exhibited a robust humoral immune response, with a mean anti-Plac1 antibody concentration of 3.71 µg/mL. In contrast, the group vaccinated with parental cell lysates demonstrated a substantially lower mean antibody titer (0.87 µg/mL), a concentration that was significantly reduced compared to the CSC lysate group. Serum from mice administered normal saline consistently yielded results below the assay’s limit of detection (Table 2), confirming that the observed antibody production was a specific response to vaccination.

**Table 2:** Quantification of anti-Plac1 antibody levels in the Sera of mice following prophylactic and therapeutic vaccination

Vaccinated mice sera	Prophylactic vaccination group Mean ± SD (µg/mL)	Therapeutic vaccination group Mean ± SD (µg/mL)
CSC lysate vaccinated mice sera	3.72 ± 0.43	BDL*
Parental cell lysate vaccinated mice sera	0.87 ± 0.15	BDL
Normal saline injected mice sera	BDL	BDL

*Below Detection limit

Additionally, in the therapeutic vaccination group, antibody levels were found to be exceedingly low, falling below the detection limit of our ELISA system.

## Discussion

Despite significant progress in treatment, breast cancer continues to be the main cause of cancer mortality in women on a global scale [1]. Tumors are composed of heterogeneous cell populations, and the treatment effectiveness may be constrained due to heterogeneity of the tumor cells. Tumors are comprised of heterogeneous cells that possess distinct characteristics, such as resistance to therapy, the ability to metastasize, the potential for differentiation, and the capability to sustain tumor growth [37]. Cancer stem cells (CSCs) are a population of tumor cells that were first characterized by Dick et al. in acute myeloid leukemia (AML). CSCs are defined by unlimited proliferation and self-renewal, and they contribute to tumorigenesis, metastasis, and the preservation of tumor heterogeneity [38–40] and are responsible for resistance to standard chemo and radiation therapy [37]. Due to this, innovative therapeutic approaches are required, specifically those that target CSCs.

CSCs express unique neoantigens and multiple tumor-associated antigens (TAA). Some lines of evidence showed that CSCs are poorly immunogenic and evade immune responses [17, 19, 20]. Due to the broad spectrum of antigens in whole CSC lysate, it seems that using CSC lysate vaccination improves immune responses and also immunological memory and may prevent tumor recurrence [25]. Therefore, in this study, we investigated the preventive and therapeutic effects of CSC lysate-based vaccination in breast cancer mouse model.

Prophylactic vaccines are given to individuals at risk of developing certain types of cancers at pre-malignant stages to prevent cancer and to reduce global cancer morbidity and mortality [41, 42]. In addition, research in this area could lead to the identification of candidate antigens for targeted therapy against cancer [18]. Whereas, therapeutic cancer vaccines are applied in late-stage malignancies and stimulate the patient’s immune system against the tumor [43]. Although several studies and clinical trials have indicated the significant effect of therapeutic cancer vaccines in harnessing cancer growth and progression [44, 45], less research has been performed regarding the effect of therapeutic CSC-based vaccines in both human and animal models [18]. Therefore, there is an urgent need to investigate novel approaches targeting CSCs as a key player in cancer progression.

The findings of our study demonstrated that CSC lysate effectively reduced tumor growth in mice for consecutive days following prophylactic vaccination compared with the lysate of parental cells and normal saline until day 30 after tumor induction. Furthermore, the CSC lysate also significantly extended the survival time of mice with established tumors. These results are in line with our previous findings [24] and Guo et al. in the syngeneic colorectal cancer model [26]. The later study showed a reduction of CD133 + and ALDH+ cells, increased NK activity, increased IFN-γ, Perforin, and Granzyme B production, and decreased TGF-β1 expression following CSC vaccine administration [26]. Similar results were also reported by other investigators in ovarian cancer model indicating significantly increased immunocyte cytotoxicities and remarkably reduced CSC counts in the CSC-vaccinated mice [46].

The most important issue in cancer-related mortality is metastasis, therefore, finding approaches that lead to the reduction of metastasis is of particular importance in cancer biology. Our results showed that prophylactic CSC-based immunization significantly reduced metastasis in the lungs and liver of 4T1 tumor-bearing mice compared to parental cell-based vaccination. Our result is in line with the Duarte et al. findings that showed preventive CSC-based vaccination reduces liver metastasis in a rat colon carcinoma syngeneic model [23]. In therapeutic vaccination, CSC-based vaccination reduced tumor size until day 15 post-tumor cell injection compared to parental cell vaccination. In terms of metastasis, a decrease in the metastasis burden in the the lungs and livers in the CSC-based vaccinated group was observed, but this decrease was not significant. The results showed that in both prophylactic and therapeutic vaccinations, tumor size and metastasis decrease, although in therapeutic vaccination, tumor size reduction is limited to the first 15 days of tumor induction. As the tumor size increases, the inhibitory effect of vaccination on tumor growth is lost.

To investigate the level of metastasis in lungs and liver in vaccinated tumor-bearing mice, we assessed the expression level of the Plac1 gene in lungs and liver of mice. Previous investigations have demonstrated that the expression of PLAC1 in normal tissues is restricted to the placenta and at much lower levels in the testis and cerebellum [47, 48]. PLAC1 is expressed in numerous types of cancer [49]. We have previously shown that Plac1 is not expressed in the tissues of normal mice, except the testis and brain, as determined by real-time PCR. Since Plac1 is not expressed in normal mouse tissues and given that 4T1 cells express this molecule [35], we recently used the Plac1 molecule to track 4T1 cells in the lungs and liver of mice [34]. In both prophylactic and therapeutic vaccinations, there was a decrease in the burden of metastasis, but in the therapeutic vaccination, this decrease was not statistically significant. Given the fact that metastasis is the main cause of mortality in breast cancer patients [50], any strategy that leads to the reduction or inhibition of metastasis will be an achievement in the field of cancer treatment. These results are following the effect of CSC vaccination on the reduction of liver metastasis in the colorectal cancer model [23]. The use of CSC-lysate-pulsed DC as a vaccination resulted in a reduction of lung metastasis in the melanoma model [22].

To detect anti-CSC and anti-parental cell antibodies in vaccinated mice, both cells were incubated with mice sera and analyzed by flow cytometry and IF. The results showed that in both prophylactic and therapeutic vaccination settings, parental cells are stained more intensely than CSCs with all examined sera, as judged by mean fluorescence intensity (MFI). Interestingly, both cells (parent and CSC) are recognized by sera from normal saline-injected mice.

According to what was presented, vaccinations led to a decrease in tumor growth and metastasis, along with enhanced survival in tumor-bearing mice. However, when analyzing the humoral immune response against CSC antigens, it was seen that the lysate of the parental cells induced a stronger humoral immune response. What justification can be given for these apparently contradictory findings? Two explanations could be considered: First, CSCs are not immunogenic but possess molecules/epitopes that, if the immune system responds against these antigens, could cause some degree of protection. In other words, it seems that, in this context, antigens with potential therapeutic function are not sufficiently immunogenic. As other researchers have pointed out not all antigens are necessarily good immunogens [51]. In some investigations, cancer stem cells have been considered as poor immunogens, and strategies are employed to enhance the immunogenicity of antigens [23]. Several studies support this issue; for example, an investigation demonstrated that CSCs downregulate MHC class I and inhibit proliferative T-cell responses and IL-2 secretion in glioblastoma and melanoma [52, 53]. In addition, CSCs suppress the activity of antigen-presenting cells, natural killer (NK) cells, and T cells by secreting tumorigenic growth factor-β (TGF-β), interleukin IL-4, IL-13, and IL-10 [53–55]. An experiment on breast cancer revealed that CSCs reduce T helper 1 response, increase IL-10 secretion, and attenuate neutrophil infiltration by overexpressing surface molecules, such as CD200 [56, 57]. There are several strategies to overcome this problem and increase the immunogenicity of CSC antigens, including; improving the delivery route of immunogens, choosing the right adjuvant, modifications in lysate preparation, and increasing the frequency or dose of vaccine. Second, both the innate and adaptive immune systems are essential for generating an effective immune response. Humoral immunity and cell-mediated immunity are two arms of the immune system [58]. Cellular immunity can be responsible for the results we obtained in our investigation. Our previous *invitro* investigation supported that CSC-derived lysate and exosomes can stimulate DC maturation and T-cell cytotoxicity, resulting in anti-tumor responses in colorectal cancer [12]. T-cell infiltration in tumors is regarded as a favorable prognostic indicator for the control of tumor growth in numerous experimental models. Elevated levels of CD4 and CD8 T cells in tumors of mice vaccinated with prostate CSC-antigen were shown by de la Luz Garcia-Hernandez et al. and this increased infiltration was associated with reduced tumor growth and enhanced survival in vaccinated mice [59, 60].

Interestingly, in the serum of tumor-bearing mice that received normal saline, there were antibodies against parental and CSC cells, which reveals that the intrinsic immune response against the tumor was activated after tumor induction. It is possible that necrotic or dying tumor cells caused such responses. Several studies have demonstrated that the immunogenicity of dying cells in the tumor microenvironment, via specific activation of the necroptotic pathway, elicits an immune response [61–63].

The ELISA data indicate that, anti-Plac1 antibody titers were significantly higher in mice vaccinated with CSCs compared with those receiving parental cell lysate. This differences in humoral response is likely attributable to the higher expression of the Plac1 molecule on 4T1-CSCs relative to parental cells. This finding is consistent with prior work by Farhangnia et al. [64], which demonstrated elevated Plac1 molecule expression in cancer stem cell-enriched populations of PC3 cell line compared to parental cells.

### Limitation

A limitation of this study is the lack of immunohistochemical staining for proliferation (e.g., Ki-67) and immune cell markers (e.g., CD3, CD11b) in murine lung and liver tissues. The inclusion of such data would have provided a more comprehensive analysis of tumor growth kinetics and the local host immune response. The other limitation of this study is that 4T1 cells in metastatic target organs were not stained with an anti-Plac1 antibody by immunohistochemistry.

## Conclusion

The study conducts a comprehensive examination of the effectiveness of preventive and therapeutic vaccinations utilizing the whole lysate of cancer stem cells (CSCs) in a mouse model of breast cancer. An important feature of the study is the comparison between CSC lysate vaccinations and parental cell lysate vaccines. The study’s findings highlight the potential of CSC-based vaccinations to substantially decrease tumor development and metastasis, therefore improving survival rates in mice. This approach marks a promising advancement in cancer immunotherapy, and this research establishes a foundation for future studies to enhance vaccine formulations and administration techniques, with the ultimate goal of applying these findings in clinical settings to greatly enhance the effectiveness of cancer treatment.

## Supplementary Information

Below is the link to the electronic supplementary material.

Supplementary Material 1

## Data Availability

The data used to support the findings of this study are available from the corresponding author on reasonable request.
