# Supplementary material for: Prophylactic and therapeutic CSC-based vaccination reduced tumor growth, metastasis and enhanced survival in mouse model of breast cancer
Source: Breast Cancer Res. 2026 Feb 19;28:63. doi: 10.1186/s13058-026-02242-7 (PMC13032256; doi:10.1186/s13058-026-02242-7)
Supplement: Supplementary file 1 — Supplementary Material 1 [file 13058_2026_2242_MOESM1_ESM.docx]

**A**

**B**

**Fig S1. Comparative evaluation of metastasis burden in Lungs and Liver of mice following A: prophylactic and B: therapeutic vaccination**. Ten randomly selected fields are analyzed by image J software and the percentage of parenchymal tissue area occupied by metastases was calculated Statistical significance is indicated as follows: *p < 0.05, **p < 0.01, ***p < 0.001 and ns: not significant.
